# Analysis of tumor markers in pleural effusion and serum to verify the correlations between serum tumor markers and tumor size, TNM stage of lung adenocarcinoma

**DOI:** 10.1002/cam4.2809

**Published:** 2019-12-27

**Authors:** Zhongqing Chen, Ying Wang, Min Fang

**Affiliations:** ^1^ Department of Clinical Laboratory Guangxi Medical University Cancer Hospital Nanning Guangxi People’s Republic of China

**Keywords:** lung adenocarcinoma, pleural effusion, serum, tumor marker

## Abstract

**Background:**

The study of tumor markers (TM) in pleural effusion (PE) was not extensive.

**Methods:**

TM in PE and serum were analyzed to determine whether TM was expressed in intrathoracic and extrathoracic tissues. To further verify the correlations between serum TM and tumor size, TNM stage of lung adenocarcinoma.

**Results:**

Serum AFP was not correlated with tumor size, T stage, N stage, and M stage (*P* > .05). Serum CEA, serum CA125, serum CA15‐3 were positively correlated with tumor size, T stage, N stage, M stage (*P* < .05). Serum CA19‐9 was not significantly correlated with tumor size and T stage (*P* > .05), but was positively correlated with N stage and M stage (*P* < .05). The levels of PE CEA, PE CA125, PE CA15‐3 were higher than those of serum CEA, serum CA125, serum CA15‐3 (all *P* < .05). The level of PE AFP was lower than that of serum AFP (*P* < .05). The level of PE CA19‐9 was not significantly different from that of serum CA19‐9 (*P* > .05). The positive rates of PE CEA and PE CA125 were higher than those of serum CEA and serum CA125 (*P* < .05). The positive rates of PE AFP, PE CA15‐3, PE CA19‐9 were not significantly different from those of serum AFP, serum CA15‐3, serum CA19‐9 (*P* > .05).PE CEA, PE CA125, PE CA15‐3 were moderately positively correlated with serum CEA, serum CA125, serum CA15‐3, respectively (*r* = 0.597; *r* = 0.46; *r* = 0.583, all *P* < .05). However, PE AFP and PE CA19‐9 were very strongly positively correlated with serum AFP and serum CA19‐9, respectively (*r* = 0.888; *r* = 0.874, all *P* < .05).

**Conclusion:**

The expression characteristics of TM in PE and serum supported the correlations between serum TM and tumor size, TNM stage of lung adenocarcinoma.

## Graphical abstract

This study found that although several TM expressed differently in different degrees in patients with lung adenocarcinoma, their expression may be in primary or metastatic lesions, or both. These expression characteristics were helpful for the diagnosis and metastasis judgment of lung adenocarcinoma. Immunohistochemistry was the golden standard for the detection of TM expression, and there were few reports on this aspect in pathology. In addition, using PE to study TM of lung adenocarcinoma may be more useful than using serum.

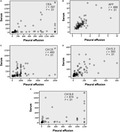

## 1 INTRODUCTION

Pleural effusion (PE) was a blood filtrate. The pathogenesis of PE was tumor invading the pleura (parietal pleura and visceral pleura), or inflammation invading the pleura. Both of these conditions can affect the production of PE by parietal pleura or the absorption of PE by visceral pleura, resulting in increased PE. The formation mechanism of PE in lung cancer may be both.^1^ PE was more common in patients with advanced lung cancer, and the formation of PE often predicted a worsening condition for lung cancer patients. However, the underlying mechanisms were still under study.^2^, ^3^, ^4^, ^5^

The expression products of lung tumors first entered PE. PE was diluted after entering the blood. Therefore, the expression products of lung tumors first appeared in PE, followed by blood. Blood and PE were in circulation. Similarly, the expression products from extrathoracic tissues first entered the blood, followed by PE. It was assumed that a certain TM was expressed only in the primary tissue of lung adenocarcinoma, but not in any metastatic tissues. The level and positive rate of this TM in PE was significantly higher than that in serum.^6^, ^7^, ^8^, ^9^, ^10^, ^11^ Since the TM in blood was only derived from PE, the correlation between PE TM and serum TM was high.^12^ The amount of blood in human body was much higher than the amount of PE. A TM expressed only in extrathoracic tissue was circulated from the blood into the PE, there may be no significant difference in the level and positive rate of PE TM and serum TM. However, the correlation between PE TM and serum TM was still high. When a TM was expressed in both intrathoracic and extrathoracic tissues, the correlation between PE TM and serum TM was low.

In our previous study,^13^ serum CEA, serum CA125, serum CA15‐3 were positively correlated with T stage, N stage, and M stage of lung adenocarcinoma. Serum CA19‐9 was positively correlated with N stage and M stage of lung adenocarcinoma. The levels of serum TM may be influenced by tumor size, T stage, N stage, M stage, and other factors, so, the correlation coefficients were low. The results may be biased. To further verify the correlations between serum TM and tumor size, TNM stage of lung adenocarcinoma, we introduced PE for lung adenocarcinoma. Since PE tended to occur in advanced patients, the number of cases was small and PE rarely tested TM items. In addition, these patients had been treated with anti‐tumor therapy, the correlations between TM and TNM stage were inaccurate. We analyzed the expression characteristics of TM in primary and metastatic tissues by comparing PE TM with serum TM. In this study, the first part would detail the correlations between serum CEA, serum AFP, serum CA125, serum CA15‐3, serum CA19‐9 and tumor size, TNM stage of lung adenocarcinoma. Tumor size and T stage represented primary tissue. N stage and M stage represented metastatic tissue. The second part would analyze serum TM and PE TM of lung adenocarcinoma. The two parts were mutually verified to further clarify the expression characteristics of TM in primary and metastatic tissues of lung adenocarcinoma.

## 2 MATERIALS AND METHODS

### 2.1 Patient information

This study was approved by the ethics committee of Guangxi Medical University Cancer Hospital with an Ethics approval number of LW2018011. This was a retrospective study that reviewed patients' TM indicators. The patients in this study consisted of two parts. The first part were 335 cases of lung adenocarcinoma patients, which were hospitalized from October 2014 to August 2017. Included patients were those who met the following criteria: (a) The pathological diagnosis was lung adenocarcinoma; (b) No antineoplastic treatment; (c) Blood samples were collected on the second day of admission to test TM; (d) There were definite tumor size and TNM stage; (e) No other malignant tumors. The second part consisted of 84 cases of lung adenocarcinoma patients who were hospitalized from October 2015 to July 2018. Included patients were those who met the following criteria: (a) The pathological diagnosis was lung adenocarcinoma; (b) PE and serum were drawn on the same day to test TM; (c) No other malignant tumors; (d) PE came from the side of lung adenocarcinoma. All cases were staged according to the 2009 seventh edition^14^, ^15^, ^16^ and 2017 eighth edition^17^, ^18^, ^19^ of the tumor‐nodes‐metastasis (TNM) classification for lung cancer. Complete and detailed information was available for all cases.

### 2.2 Tumor marker assay

The tests were completed on the day of blood drawn and PE drawn to avoid attenuation. All assays were performed according to instrument and reagent specifications. CEA, AFP, CA125, CA15‐3, and CA19‐9 were measured using ARCHITECTi2000SR automatic electrochemical luminescence instrument and matching reagents (Abbott Laboratories, Chicago). TM levels above the following values were considered abnormal: CEA, 5ng/ml; AFP 30ng/ml; CA125, 35ng/ml; CA15‐3, 30ng/ml; CA19‐9, 37ng/ml.

### 2.3 CT scanning

Chest CT scans were performed using a Siemens somatom sensation 64‐slice CT or a GE Discovery CT 750 HD. Scanning scheme: Ask the patients to hold their breath for a scan after inhaling. Using CARE DOSE technology, 120 kV, pitch 1.000, layer thickness 5 mm; lung window (window width 1500 HU, window position −450 HU), mediastinum window (window width 400 HU, window position 40 HU), layer thickness 1 mm, interval 1 mm. Films were read by two experienced radiologists.

### 2.4 Statistical methods

Data were statistically analyzed using IBM SPSS21.0 software. The significance level was *P* < .05 (two tailed). Based on the normality test (skew and kurtosis coefficients), TM levels did not obey normal distribution. The levels between groups were compared by nonparametric rank sum test, Mann‐Whitney U test. The positive rates between groups were compared by Chi‐square test (*χ* ^2^). The correlation was analyzed by Kendall's tau‐b correlation analysis. The levels of TM may be influenced by tumor size, T stage, N stage, M stage, and other factors, so, the correlation coefficients between tumor size, TNM stage, and TM levels were low. The correlation analysis in part 1 was based on *P* < .05, not the correlation coefficient. In part 2, the correlation coefficients were still used to determine the correlation. Correlation coefficient grading: 0.8‐1.0 Very strong correlation, 0.6‐0.8 strong correlation, 0.4‐0.6 moderate correlation, 0.2‐0.4 weak correlation, 0.0‐0.2 very weak correlation or no correlation.

## 3 RESULTS

### 3.1 Patient population and characteristics

Table 1 showed the patient population and characteristics. Part 1 was the information of 335 cases of lung adenocarcinoma. They were all first diagnosed and had not been treated with any anti‐tumor therapy, such as surgery, chemotherapy, radiotherapy, biological therapy, endocrine therapy, Chinese medicine treatment, hyperthermia, and radiofrequency ablation therapy. Since decreased TM levels in patients with effective treatment will affect the correlation analysis between TNM stage and TM levels. In this cohort, there were 205 males (61.2%) and 130 females (38.8%). The average age was 57 years (28‐86 years old). The primary tumor size of the lungs was 0.8‐13 cm. This part of the patients had detailed TNM stage data, T_1_ 83 (24.8%), T_2_ 157 (46.9%), T_3_ 58 (17.3%), T_4_ 37 (11%); N_0_ 107 (31.9%), N_1_ 41 (12.2%), N_2_ 114 (34%), N_3_ 73 (21.8%); M_0_ 203 (60.6%), M_1_ 132 (39.4%).

**Table 1:** Characteristics of the study subjects

Parameters	Part 1		Part 2	
	No.	%	No.	%
Gender				
Male	205	61.2	51	60.7
Female	130	38.8	33	39.3
Age (year)				
Range	28‐86		30‐75	
Mean	57		56.6	
Tumor size (cm)	0.8‐13		—	
T stage				
T_1_	83	24.8	7	8.3
T_2_	157	46.9	10	11.9
T_3_	58	17.3	9	10.7
T_4_	37	11	36	42.9
Tx	0	0	22	26.2
N stage				
N_0_	107	31.9	5	6
N_1_	41	12.2	4	4.8
N_2_	114	34	22	26.2
N_3_	73	21.8	31	36.9
Nx	0	0	22	26.2
M stage				
M_0_	203	60.6	0	0
M_1_	132	39.4	84	100

Part 2 was the information of 84 cases of lung adenocarcinoma all in stage IV and had been treated with anti‐tumor agents. In this cohort, there were 51 (60.7%) males and 33 females (39.3%). The average age was 56.6 years (30‐75 years old). Some patients did not have detailed TNM stage data, T_1_ 7 (8.3%), T_2_ 10 (11.9%), T_3_ 9 (10.7%), T_4_ 36(42.9%), T*_x_* 22 (26.2%); N_0_ 5 (6%), N_1_ 4 (4.8%), N_2_ 22 (26.2%), N_3_ 31 (36.9%), Nx 22 (26.2%); M_1_ 84 (100%).

### 3.2 Correlation between tumor size, TNM stage, and serum tumor markers levels in part 1

Table 2 showed the serum TM of 335 cases of lung adenocarcinoma. First, the minimum, maximum, median, positive rates of serum TM were analyzed, and then the correlations between serum TM and tumor size, TNM stage were analyzed. The lowest values of CEA, AFP, CA125, CA15‐3, and CA19‐9 were lower than the upper line of normal value. The highest values of CEA, AFP, CA125, CA15‐3, and CA19‐9 were higher than the upper line of normal value. The median of CEA was higher than the upper line of normal value, the median of AFP, CA125, CA15‐3, and CA19‐9 were lower than the upper line of normal value. The positive rates were ranked as CEA>CA125>CA15‐3>CA19‐9>AFP. CEA was positively correlated with tumor size, T stage, N stage, M stage (*r* = 0.146; *r* = 0.218; *r* = 0.349; *r* = 0.358, all *P* < .05). CA125 was positively correlated with tumor size, T stage, N stage, M stage (*r* = 0.219; *r* = 0.298; *r* = 0.398; *r* = 0.358, all *P* < .05). CA15‐3 was positively correlated with tumor size, T stage, N stage, M stage (*r* = 0.197; *r* = 0.254; *r* = 0.325; *r* = 0.282, all *P* < .05). CA19‐9 was not significantly correlated with tumor size and T stage (*r* = 0.063; *r* = 0.061, all *P* > .05). CA19‐9 was positively correlated with N stage and M stage (*r* = 0.118; *r* = 0.145, all *P* < .05). There were no correlations between AFP and tumor size, T stage, N stage, M stage (*r* = −0.050; *r* = −0.052; *r* = −0.026; *r* = 0.068, all *P* > .05).

**Table 2:** The range, median, positive rates, and correlation analysis of serum tumor markers and TNM stage of lung adenocarcinoma in part 1

Parameters	CEA	AFP	CA125	CA15‐3	CA19‐9
Range (ng/mL)	0.65‐1000	0.60‐89.66	0.60‐2103	5.24‐300	0.23‐1000
Median (ng/mL)	6.82	2.66	20.28	19.93	14.35
Positive rate (%)	62.4	2.1	36.7	28.4	21.5
Correlation					
Tumor size					
*r*	.146	−.050	.219	.197	.063
*P*	.000	.173	.000	.000	.087
T stage					
*r*	.218	−.052	.298	.254	.061
*P*	.000	.213	.000	.000	.146
N stage					
*r*	.349	−.026	.398	.325	.118
*P*	.000	.534	.000	.000	.004
M stage					
*r*	.358	.068	.358	.282	.145
*P*	.000	.127	.000	.000	.001

### 3.3 Correlation between PE tumor markers levels and serum tumor markers levels in part 2

Table 3 showed the PE TM and serum TM of 84 cases of lung adenocarcinoma. First, the minimum, maximum, median, positive rates of PE TM, and serum TM were analyzed, and then the correlations between serum TM and PE TM were analyzed.

**Table 3:** Tumor markers levels in pleural effusion and serum of lung adenocarcinoma in part 2

Parameters	CEA	AFP	CA125	CA15‐3	CA19‐9
Range (ng/mL)					
PE	0.5‐1500	0.53‐8.61	5.6‐5000	1.2‐800	0‐1200
Serum	0.5‐1500	1.29‐12.16	5.7‐1340	4.14‐300	0.1‐1200
Median					
PE (ng/mL)	100.00	1.57	600.00	64.23	13.60
Serum (ng/mL)	18.95	2.80	98.05	36.30	18.20
*z*	−4.397	−6.986	−7.735	−2.000	−0.814
*P*‐value	.000	.000	.000	.045	.416
Positive rate					
PE (%)	88.1	0.0	95.2	66.7	34.5
Serum (%)	72.6	0.0	85.7	60.7	32.1
*χ* ^2^	6.373	—	4.421	0.643	0.107
*P*‐value	.012	—	.035	.422	.743
Correlation					
*r*	0.597	0.888	0.460	0.583	0.874
*P*‐value	.002	.000	.024	.003	.000

In both PE and serum, the lowest values of CEA, AFP, CA125, CA15‐3, and CA19‐9 were lower than the upper line of normal value, the maximum values of CEA, CA125, CA15‐3, and CA19‐9 were higher than the upper line of normal value, the maximum value of AFP was lower than the upper line of normal value. The median of CEA, CA125, and CA15‐3 were higher than the upper line of normal value, the median of AFP and CA19‐9 were lower than the upper line of normal value. The levels of PE CEA, PE CA125, and PE CA15‐3 were higher than those of serum CEA, serum CA125, serum CA15‐3, respectively (*z* = −4.397; *z* = −7.735; *z* = −2.000, all *P* < .05). The level of PE AFP was lower than that of serum AFP (*z* = −6.986, *P* < .05). The level of PE CA19‐9 was not significantly different from that of serum CA19‐9 (*z* = −0.814, *P* > .05).

The positive rates were ranked as CA125>CEA>CA15‐3>CA19‐9>AFP in both PE and serum. The positive rates of PE CEA and PE CA125 were higher than those of serum CEA, serum CA125, respectively. The positive rates of PE AFP, PE CA15‐3, and PE CA19‐9 were not significantly different from those of serum AFP, serum CA15‐3, serum CA19‐9, respectively (*z* = 0.000; *z* = 0.643; *z* = 0.107, all *P* > .05).

**Figure 1:**
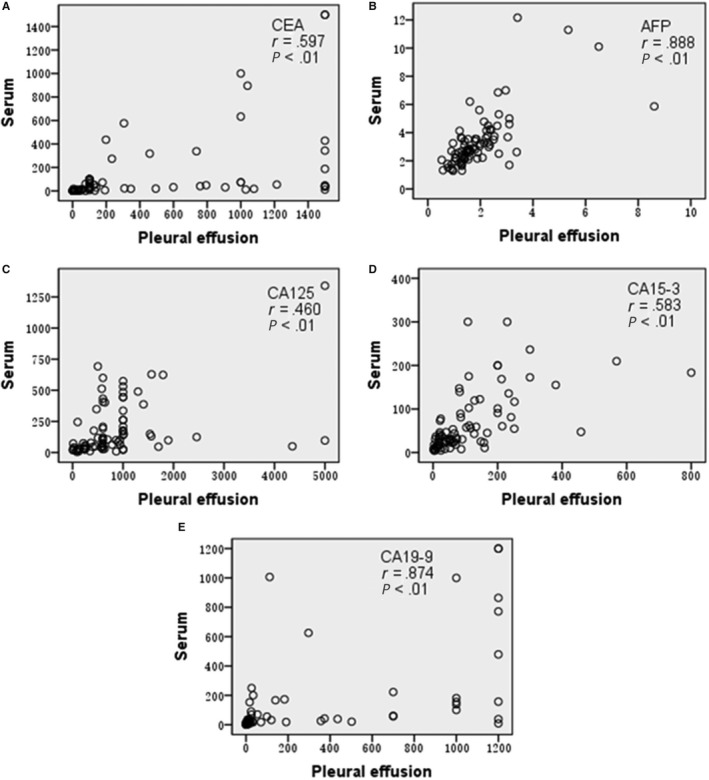
A, Correlation between pleural effusion (PE) CEA and serum CEA; B, Correlation between PE AFP and serum AFP; C, Correlation between PE CA125 and serum CA125; D, Correlation between PE CA15‐3 and serum CA15‐3; E, Correlation between PE CA19‐9 and serum CA19‐9

PE CEA, PE CA125, PE CA15‐3 were moderately positively correlated with serum CEA, serum CA125, serum CA15‐3, respectively (*r* = 0.597; *r* = 0.46; *r* = 0.583, all *P* < .05). PE AFP, PE CA19‐9 were very strongly positively correlated with serum AFP and serum CA19‐9, respectively (*r* = 0.888; *r* = 0.874, all *P* < .05). Figure 1.

## 4 DISCUSSION

Exclude patients without tumor size data, only 335 cases of lung adenocarcinoma were studied in part 1. It was noteworthy that the conclusions were consistent with the previous 424 cases of lung adenocarcinoma.^13^ Tumor size largely determines T stage. The correlations between TM levels and tumor size were consistent with the correlations between TM levels and T stage. The production of PE may be due to inflammation or tumors. Normal cells and certain benign diseases can only synthesize or secrete CEA in small amounts.^20^ CA125 levels were significantly higher in malignant PE than in benign PE.^21^, ^22^ The positive rate of PE CEA was 88.1%, the positive rate of PE CA125 was 95.2%. The causes of PE and elevated PE TM were still by tumor. We compared serum TM and PE TM in part 2. The positive rates were consistently ranked as CA125>CEA>CA15‐3>CA19‐9>AFP in both PE and serum. PE TM was moderately and above positively correlated with serum TM.PE CA19‐9 and PE AFP were highly correlated with serum CA19‐9 and serum AFP, suggesting that CA19‐9 and AFP were expressed in intrathoracic or extrathoracic tissues. PE CEA, PE CA125, PE CA15‐3 were positively correlated with serum CEA, serum CA125, serum CA15‐3, suggesting that CEA, CA125, CA15‐3 were expressed in both intrathoracic and extrathoracic tissues.

AFP was a TM of hepatocellular carcinoma and was irrelevant to lung adenocarcinoma.^23^ In part 2, the positive rates of PE AFP and serum AFP were all 0%. PE AFP level was significantly lower than that of serum AFP. There was a very strong correlation between PE AFP and serum AFP. AFP may be only expressed in extrathoracic tissues, which supported there were no significant correlations between serum AFP level and tumor size, T stage, N stage, M stage in part 1.

CEA was the antibody to be tumor‐cell specific. CEA was widely recognized as a TM with high sensitivity and specificity for lung cancer,^24^, ^25^, ^26^ especially lung adenocarcinoma.^21^, ^27^, ^28^, ^29^ CEA also has a good performance in the identification of benign and malignant PE.^30^, ^31^ PE CA125 of carcinomatous pleuritis was produced by not only carcinoma cells but also activated mesothelial cells.^32^ In part 2, the expression of CEA and CA125 in serum and PE were similar. The median and positive rates of PE CEA (100,88.1%), PE CA125 (600,95.2%), serum CEA (18.95,72.6%), serum CA125 (98.05,85.7%) were at high levels. The levels and positive rates of PE CEA and PE CA125 were significantly higher than those of serum CEA and serum CA125, respectively. Moderate positive correlations were observed between PE CEA, PE CA125 and serum CEA, serum CA125, similar to another report.^12^ CEA and CA125 were probably expressed in both intrathoracic and extrathoracic tissues, and the expression levels in intrathoracic tissues were higher than that in extrathoracic tissues. So, in part 1, serum CEA and serum CA125 were positively correlated with tumor size, T stage, N stage, M stage.

CA15‐3 was commonly used in diagnosis and prognosis of breast cancer.^33^ High PE CA15‐3 levels indicated inflammatory disease or malignant tumor.^34^ Higher PE CA15‐3 levels were observed in malignant PE with positive cytology.^35^ PE CA15‐3 showed significantly high specificity in malignant PE.^36^, ^37^, ^38^, ^39^ In part 2, the level of PE CA15‐3 was higher than that of serum CA15‐3, the positive rate of PE CA15‐3 was not significantly different from serum CA15‐3. PE CA15‐3 were moderately positively correlated with serum CA15‐3. The positive rates of PE CA15‐3 and serum CA15‐3 (66.7%, 60.7%) were lower than those of CEA (88.1%, 72.6%) and CA125 (95.2%, 85.7%). CA15‐3 may be expressed in both intrathoracic and extrathoracic tissues, but was less expressed than CEA and CA125. So, in part 1, serum CA15‐3 was significantly positively correlated with tumor size, T stage, N stage, M stage.

Some studies suggested that the level of PE CA19‐9 in malignant PE and benign PE were similar,^6^ CA19‐9 was significantly increased in lung adenocarcinoma‐associated malignant PE compared to benign PE,^40^, ^41^ PE CA19‐9 was hyposensitive to lung adenocarcinoma.^42^ While other studies reported PE CA19‐9 and PE CA125 in bronchoalveolar lavage fluid of lung cancer were more useful markers than PE CA15‐3.^43^ So, CA19‐9 was controversial in identifying benign and malignant PE. In part 2, PE CA19‐9 was strongly positively correlated with serum CA19‐9, indicating that CA19‐9 was only expressed in intrathoracic or extrathoracic tissues. The blood volume of human body was much higher than the volume of PE in the thoracic cavity. When CA19‐9 enters PE from the blood, the concentration of CA19‐9 decreased insignificantly. The level and positive rate of PE CA19‐9 were not significantly different from those of serum CA19‐9. CA19‐9 maybe expressed in extrathoracic tissues, may be associated with the metastasis of lung adenocarcinoma. So, in part 1, serum CA19‐9 was not significantly correlated with tumor size and T stage, but serum CA19‐9 was significantly positively correlated with N stage and M stage.

## 5 CONCLUSIONS

The expression characteristics of TM in PE and serum supported the correlations between serum TM and tumor size, TNM stage of lung adenocarcinoma. Serum CEA, serum CA125, and serum CA15‐3 were positively correlated with tumor size, T stage, N stage, M stage of lung adenocarcinoma. Serum CA19‐9 was positively correlated with N stage, and M stage of lung adenocarcinoma, but not correlated with tumor size and T stage. Serum AFP was not correlated with tumor size, T stage, N stage, and M stage of lung adenocarcinoma.

## CONFLICTS OF INTEREST

The authors have no conflicts of interest to declare.

## AUTHOR CONTRIBUTIONS

ZQC, YW and MF conceived and designed the project. ZQC and YW collected the clinical specimens and data. ZQC, YW and MF performed the statistical analysis and wrote the manuscript. ZQC, YW and MF contributed to the writing and critical reading of the paper. All authors read and approved the final manuscript.

## ETHICAL APPROVAL

This study was approved by the ethics committee of the Guangxi Medical University Cancer Hospital with an ethics approval number of LW2018011.

## CONSENT FOR PUBLICATION

Not applicable.

## Data Availability

The datasets used during the current study were available from the corresponding author on reasonable request.
